# Adhesion between *P. falciparum* infected erythrocytes and human endothelial receptors follows alternative binding dynamics under flow and febrile conditions

**DOI:** 10.1038/s41598-020-61388-2

**Published:** 2020-03-11

**Authors:** Pedro Lubiana, Philip Bouws, Lisa Katharina Roth, Michael Dörpinghaus, Torben Rehn, Jana Brehmer, Jan Stephan Wichers, Anna Bachmann, Katharina Höhn, Thomas Roeder, Thorsten Thye, Thomas Gutsmann, Thorsten Burmester, Iris Bruchhaus, Nahla Galal Metwally

**Affiliations:** 1 Bernhard Nocht Institute for Tropical Medicine, Hamburg, Germany; 2 Molecular Physiology Department, Zoological Institute, Christian-Albrechts University Kiel, Kiel, Germany; 3 Division of Biophysics, Research Center Borstel, Leibniz-Center for Medicine and Biosciences, Borstel, Germany; 4 Zoological Institute, Department of Molecular Physiology, Hamburg University, Hamburg, Germany; 5 Department of Biology, University of Hamburg, Hamburg, Germany

**Keywords:** Parasite immune evasion, Parasite biology

## Abstract

Characterizing the adhesive dynamics of *Plasmodium falciparum* infected erythrocytes (IEs) to different endothelial cell receptors (ECRs) in flow is a big challenge considering available methods. This study investigated the adhesive dynamics of IEs to five ECRs (CD36, ICAM-1, P-selectin, CD9, CSA) using simulations of *in vivo*-like flow and febrile conditions. To characterize the interactions between ECRs and knobby and knobless IEs of two laboratory-adapted *P. falciplarum* isolates, cytoadhesion analysis over time was performed using a new tracking bioinformatics method. The results revealed that IEs performed rolling adhesion exclusively over CD36, but exhibited stationary binding to the other four ECRs. The absence of knobs affected rolling adhesion both with respect to the distance travelled by IEs and their velocity. Knobs played a critical role at febrile temperatures by stabilizing the binding interaction. Our results clearly underline the complexity of the IE-receptor interaction and the importance of knobs for the survival of the parasite at fever temperatures, and lead us to propose a new hypothesis that could open up new strategies for the treatment of malaria.

## Introduction

Cytoadhesion of *Plasmodium falciparum* to human endothelial cell receptors (ECRs) causes complications and deaths following malaria infection. In 2018, 405,000 malaria-related deaths were registered (61% of which were of children younger than 5 years old)^1^. Cytoadhesion leads to accumulation of infected erythrocytes (IEs) within the microvascular bed of vital organs such as the brain, lungs, and kidneys. Death can eventually occur due to decreased blood supply and organ failure^2,3^. Cytoadhesion results from interactions between members of the *P. falciparum* erythrocyte membrane protein 1 (*Pf*EMP1) family and different ECRs^4–8^. About 60 *var* genes per parasite genome encode *Pf*EMP1 family members. Only one *Pf*EMP1 variant is located on the membrane of IEs at the trophozoite stage, but the corresponding *var* gene is already expressed at the ring stage in a mutually exclusive pattern^9^. The most studied interaction partners are the ECRs CD36, intracellular adhesion molecule 1 (ICAM-1), endothelial protein C receptor, and chondroitin sulfate A (CSA)^6,7^. In general, *Pf*EMP1 molecules cluster on nanoscale protrusions, called knobs, located on the membrane of IEs. Knobs consist of various submembranous structural proteins, predominantly knob-associated histidine-rich protein (KAHRP)^10^. KAHRP contains several binding domains that interact with both parasite and host factors. Knobs begin to appear on the surface of IEs at 16 h post-invasion (hpi). The density of knobs increases from 20 to 60/µm^2^ with parasite development from the trophozoite stage to the schizont stage respectively^11^. The presence of knobs on the surface of IEs leads to increase their stiffness, which in turn increases their binding ability^11,12^.

Many aspects of IE-ECR interactions have not been clarified. The leukocyte cytoadhesion model of recruitment and extravasation to the site of inflammation has been proposed. This model states that adhesion of leukocytes to various ECRs during inflammation can be divided into three main phases called tethering, rolling, and immobilization. During the tethering phase, leukocytes contact three selectin receptors. Thereafter, rolling movement is enhanced by α_4_β_7_ and α_4_β_1_. Finally, firm immobilization to ICAM-1 or ICAM-2 occurs^13–15^. It is uncertain whether IEs use the same cytoadhesive mechanics as leukocytes. Controversial data regarding IE-ECR cytoadhesive dynamics under flow conditions have been reported. For example, IE-ICAM-1 adhesive mechanics were described by some authors as rolling adhesion and by others as static binding, similar to IE-CD36 adhesive mechanics^16–19^.

Cytoadhesive dynamics are dependent on cell properties, ligand-receptor bond dynamics, and flow conditions^14,20^. The membranes of IEs are 10-fold stiffer than those of non-IEs due to the presence of knobs. In addition, trophozoite-stage IEs are biconcave and parasites localize to the periphery of these cells, while schizont-stage IEs are spherical and parasites localize throughout these cells^21,22^. These changes alter the adhesive properties of IEs^20,23^. In terms of adhesive bond dynamics between IEs’ ligands and ECRs, there are two types of bonds, termed slip and catch bonds, which are formed by non-covalent interactions. Life time of slip bonds is decreased in the presence of an external tensile force. Conversely, that of catch bonds increase with increasing external force^24^. However, catch bonds can decrease with very high external force and this is called catch-slip bonds^25^. In terms of flow conditions, the arterial and venous vasculature is exposed to different shear stresses, which correspond with differences in the blood flow rate/velocity between arteries and veins. Normal physiological shear stress ranges from 10 to 70 dyn/cm^2^ in arteries, but from 1 to 6 dyn/cm^2^ in veins^26^. Shear stress affects the stabilities of different types of bonds, which in turn affects IE-ECR adhesive dynamics^24–28^.

A cardinal host response to *P. falciparum* infection is fever at irregular intervals, and IEs are important in the febrile state. Fever during infection modulates the immune response to the pathogen. *In vitro* experiments demonstrated the effect of fever on protein expression and parasite survival^29,30^. However, the effects of fever on the adhesive dynamics and binding affinities of IEs are poorly described in the literature. This study describes the cytoadhesive behavior of IEs to a variety of ECRs (CD36, ICAM-1, P-selectin, CD9, and CSA). We simulated the dynamic forces acting on IEs in the human circulation using a laminar flow system and various shear stresses. We also investigated the effect of febrile temperature (40 °C) on the binding dynamics and affinities of IEs to different ECRs. The results led us to formulate a new hypothesis regarding IE-ECR interactions during infection.

## Results

### Experimental setup

**Figure 1:**
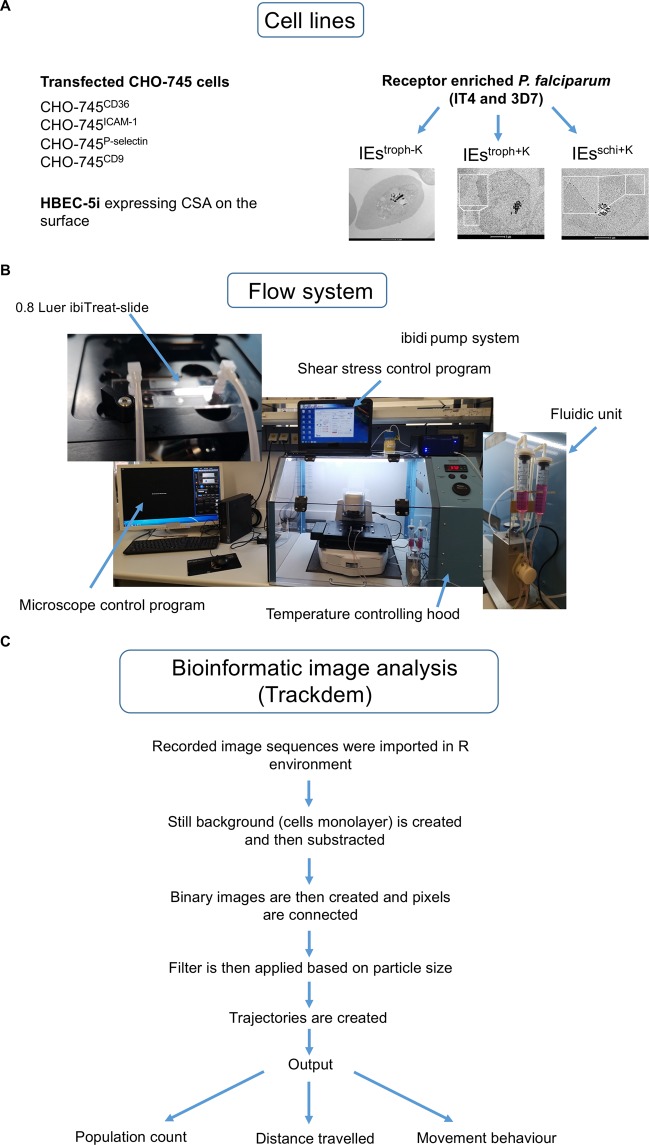
Scheme for the analysis processes. Cell lines used in this study. (**A**) IEs^troph−K^ stands for knobless trophozoites (85–100% without knobs), IEs^troph+K^ stands for knobby trohozoites (99–100% with knobs) and IEs^schi+K^ stands for knobby schizonts (99–100% with knobs). Flow system for controlling shear stress as well as temperature. (**B**) Bioinformatics scheme for tracking analysis (**C**).

Endothelial cytoadhesion and the binding capacities of various laboratory strains and patient isolates of *P. falciparum* have been mainly investigated under static conditions^6,31,32^. This is classically achieved by directly incubating IEs with cells or recombinant receptors of interest^8,16,33,34^. Finally, adherent IEs are counted manually. These experiments demonstrated that laboratory-adapted trophozoite-stage IT4 and 3D7 isolates show different binding capacities to various ECRs under static conditions^31,33^. The main objective of this study was to characterize the binding behavior of erythrocytes infected with *P. falciparum* to the ECRs CD36, ICAM-1, P-selectin, CD9, and CSA under controlled flow and temperature conditions. For this purpose, we used transfected CHO cells expressing GFP fusions of the receptors of interest on the cell surface^33^. *P. falciparum* populations with increased binding for ICAM-1, P-selectin and CD9 receptors were obtained by performing at least seven rounds of enrichment, as described previously^33^. Whereas the IEs populations used for characterizing the binding over CD36 were obtained from the starting cultures without any enrichment, since over 80% of *Pf*EMP1 proteins contain CIDRα2-6 domains, which are involved in CD36 binding^35^. All the IEs populations were tested for the presence or absence of knobs on their cell surfaces by scanning electron microscopy (SEM) (Fig. 1A). The HBEC-5i cell line was used as a model of endothelial cell cytoadhesion to CSA. Transcriptome profiling showed that IEs populations enriched for binding to HBEC-5i showed exclusive expression of *var2csa* and the HBEC-5i enriched population bind exclusively to CSA on the surface of HBEC-5i cells (Fig. S6A)^36^. A laminar flow system was used to imitate the hydrodynamic forces exerted on IEs in blood microvessels (Fig. 1B). In all experiments, different wall shear stresses (6, 4, 3, 2, 1.5, and 0.9 dyn/cm^2^) were applied, which resemble the range of shear stress exerted on ECs lining blood microvessels^26,37^. Each magnitude of shear stress was applied for 10 min, except for 0.9 dyn/cm^2^, which was applied for 30 min. Bioinformatics image analysis was performed using the trackdem R package^38^. The IEs number was adjusted to 1 × 10^7^ before the start of each experiment. Individual IEs were tracked through the entire sequence of images, and then trajectories were constructed and plotted over the static background. The accumulated distance traveled by IEs was defined as the length of each trajectory during the entire 10 min tracking time and was obtained from the output of trackdem analysis (Fig. 1C).

### Binding behavior of trophozoite-stage IEs to CHO-745^CD36^ cells

**Figure 2:**
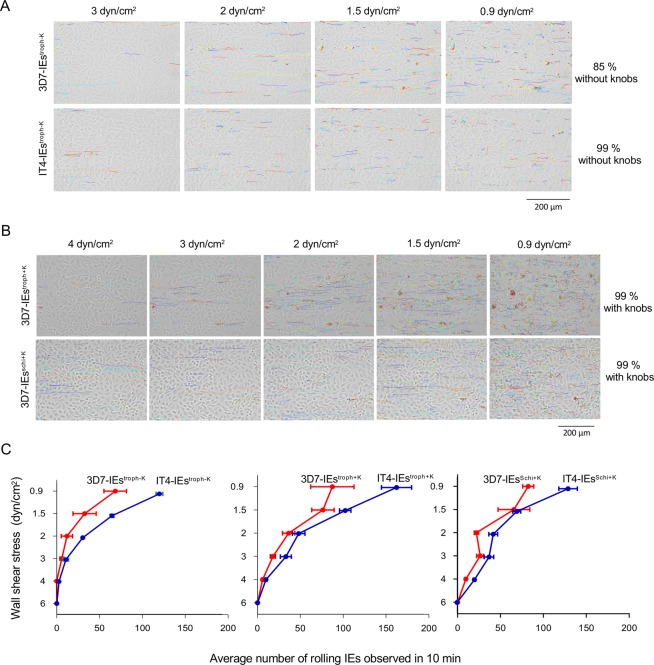
Cell trajectories showing the rolling binding behavior of IEs to CHO-745^CD36^. Trajectories of 3D7-IEs^troph−K^ and IT4-IEs^troph−K^. (**A**) Trajectories of 3D7-IEs^troph+K^ and 3D7-IEs^schi+K^ under various shear stresses. (**B**) Average number of rolling IEs at applied shear stresses. (**C**) Images were analyzed using the trackdem (0.4.2) R package^38,58^. The static cell background was subtracted from all images and then moving particles (i.e., IEs) were detected. The Shiny R package^38,58^ was used with thresholding of 0.1. The *PixelRange* of moving particles was set to 30–500. Individual trajectories were subsequently reconstructed based on a minimum present *(incThres*) equal to six frames (i.e., 30 sec). Finally, trajectories were plotted over the still background. Examples of trajectories are shown in S1 and S2 Movies.

A sequential binding pattern was observed upon exposure of knobless trophozoite-stage IEs of the two isolates IT4 and 3D7 (IT4-IEs^troph−K^ and 3D7-IEs^troph−K^, respectively) to CHO-745 cells expressing CD36 (CHO-745^CD36^). IEs were initially captured by cells and were then tethered to cells via flipping binding. IT4-IEs^troph−K^ and 3D7-IEs^troph−K^ exhibited no or very low adhesion at 6 and 4 dyn/cm^2^, meaning no trajectories were created. This reflects the inability of IEs to be captured from the flow under high shear stress. Tracking of knobless IEs of the two isolates at 3 dyn/cm^2^ revealed generation of a few trajectories (Fig. 2A,C and S1 Movie). Lowering the shear stress to 2 dyn/cm^2^ increased the number of trajectories (12 ± 6 tracks/0.2 mm^2^ for 3D7-IEs^troph−K^ and 31 ± 1 tracks/0.2 mm^2^ for IT4-IEs^troph−K^) (S2 Movie). The number of trajectories was even higher at 1.5 dyn/cm^2^ (32 ± 13 tracks/0.2 mm^2^ for 3D7-IEs^troph−K^ and 66 ± 2 tracks/0.2 mm^2^ for IT4-IEs^troph−K^) and highest at 0.9 dyn/cm^2^ (68 ± 13 tracks/0.2 mm^2^ for 3D7-IEs^troph−K^ and 124 ± 4/0.2 tracks/mm^2^ for IT4-IEs^troph−K^) (Fig. 2A,C).

### Binding behavior of knobless/knobby, trophozoite-stage IEs to CHO-745^CD36^ cells

Knobby trophozoite-stage IEs of the two isolates (IT4-IEs^troph+K^ and 3D7-IEs^troph+K^, respectively) were captured from the flow and tethered to CHO-745^CD36^ cells at high shear stress (4 dyn/cm^2^) in contrast to IEs^troph−K^. Trajectories of IEs^troph+K^ could be constructed at 4 dyn/cm^2^ (an example for 3D7-IEs^troph+K^ is shown in Fig. 2B), which was not the case for IEs^troph−K^. The number of trajectories was 6 ± 1 tracks/0.2 mm^2^ for 3D7-IEs^troph+K^ and 10 ± 1/0.2 mm^2^ for IT4-IEs^troph+K^ (Fig. 2C). At 3 dyn/cm^2^, longer trajectories (>30 µm) compared with those generated by IEs^troph−K^ were observed (Fig. 2A,B; S3 Movie). The number of trajectories was 18 ± 2 tracks/0.2 mm^2^ for 3D7-IEs^troph+K^ and 34 ± 6 tracks/0.2 mm^2^ for IT4-IEs^troph+K^ (Fig. 2C). The number of trajectories was increased at 2 dyn/cm^2^ (Fig. 2C), with 36 ± 7 tracks/0.2 mm^2^ for 3D7-IEs^troph+K^ and 49 ± 7 tracks/0.2 mm^2^ for IT4-IEs^troph+K^. The number of trajectories was even higher at 1.5 and 0.9 dyn/cm^2^ (76 ± 13 and 87 ± 25 tracks/0.2 mm^2^ for 3D7-IEs^troph+K^ and 105 ± 6 and 167 ± 18 tracks/0.2 mm^2^ for IT4-IEs^troph+K^, respectively), although these trajectories were shorter (10–30 µm) (Fig. 2B).

### Binding behavior of schizont-stage IEs to CHO-745^CD36^ cells

To compare binding behavior between the trophozoite and schizont stages, experiments were performed using knobby schizont-stage IEs (36–44 hpi) of the IT4 and 3D7 isolates (IT4-IEs^schi+K^ and 3D7-IEs^schi+K^, respectively). As an example, 3D7-IEs are shown in Fig. 2B. Similar to 3D7-IEs^troph+K^, 3D7-IEs^schi+K^ were captured from the flow by CHO-745^CD36^ cells at 4 dyn/cm^2^. Lowering the shear stress increased the number of trajectories. Specifically, the number of trajectories at 4, 3, 2, 1.5, and 0.9 dyn/cm^2^ was 9 ± 1, 26 ± 4, 22 ± 2, 65 ± 18, and 82 ± 6 tracks/0.2 mm^2^ for 3D7-IEs^troph+K^, and 20 ± 1, 38 ± 5, 43 ± 5, 71 ± 4, and 132 ± 10 tracks/0.2 mm^2^ for IT4-IEs^troph+K^, respectively (Fig. 2C and S4 Movie).

### Effects of the presence of knobs and the parasite stage on the distance traveled by and velocity of 3D7-IEs rolling over CHO-745^CD36^ cells

**Figure 3:**
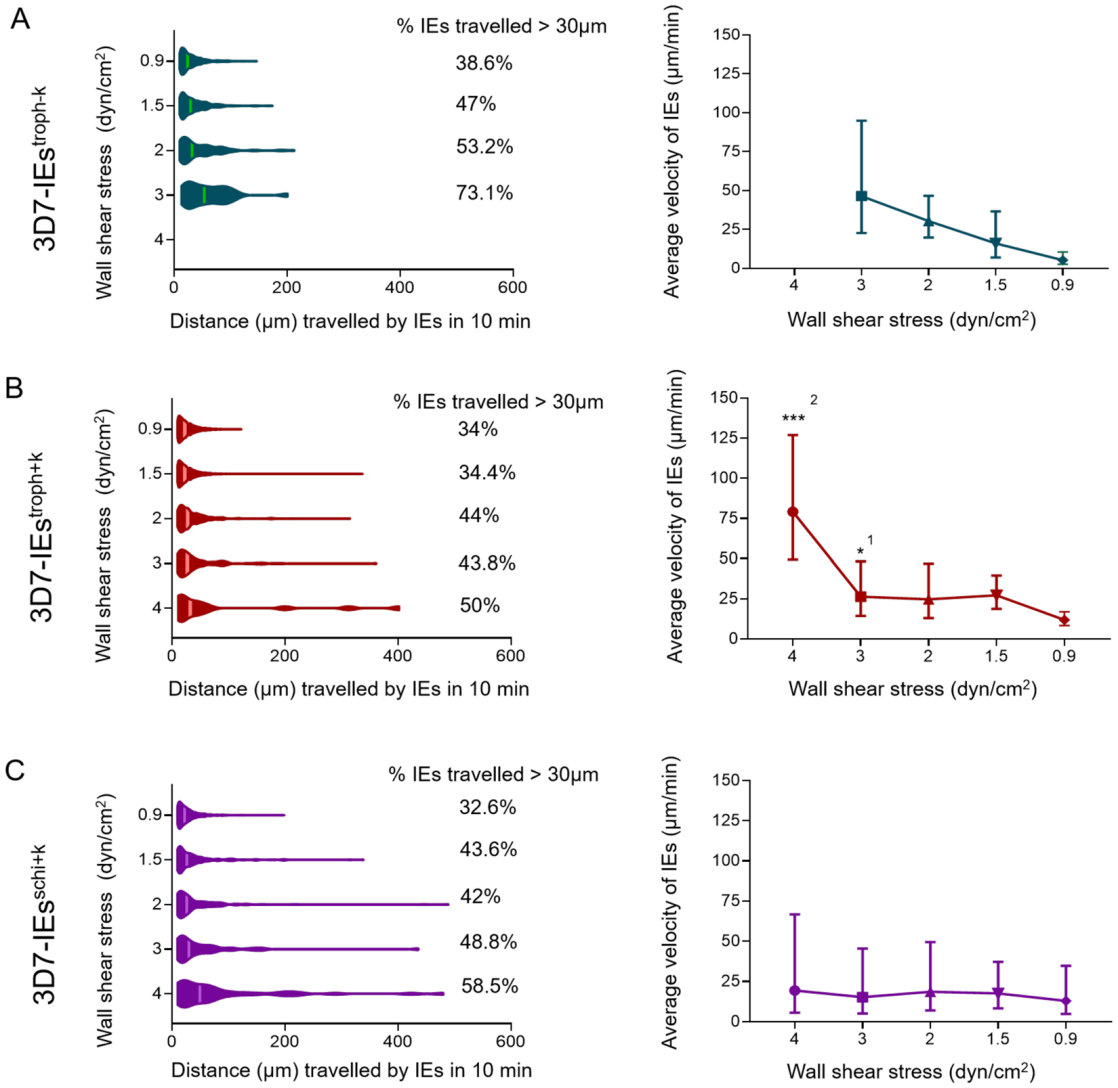
Tracking of 3D7-IEs over CHO-745^CD36^ cells. Knobless and knobby trophozoite-stage 3D7-IEs (3D7-IEs^troph−K^ (**A**), 3D7-IEs^troph+K^ (**B**) and knobby schizont-stage 3D7-IE^schi+K^ (**C**)) were analyzed. The distance traveled and the average velocity of IEs were calculated using the trackdem R package^38,58^. The distance traveled by each IE was divided by the individual contact time with cells to calculate velocity (n = 20). Significance was assessed using the Kolmogorov-Smirnov test. *p < 0.01, ***p < 0.0001 (^1^statistical comparison of 3D7-IEs^troph−K^ and 3D7-IEs^troph+K^, ^2^statistical comparison of 3D7-IEs^troph+K^ and 3D7-IEs^schi+K^).

At 3 dyn/cm^2^, 3D7-IEs^troph−K^ traveled a minimum distance of 13.43 µm and a maximum distance of 201 µm, with a geometric mean of 48 µm (Fig. 3A). At 2 dyn/cm^2^, 3D7-IEs^troph−K^ traveled a minimum distance of 10 µm and a maximum distance of 212 µm, with a geometric mean of 34 µm. At 1.5 and 0.9 dyn/cm^2^, the minimum distance traveled remained the same (10 µm), while the maximum distance traveled decreased to 174 and 146 µm, respectively. The presence of knobs on the surface of 3D7-IEs affected the distance traveled. Specifically, the maximum distance traveled by 3D7-IEs^troph+K^ was 330–400 µm at 4, 3, 2, and 1.5 dyn/cm^2^, while the minimum distance traveled was 11.5 µm at 4 dyn/cm^2^ and 10 µm at all other shear stresses (Fig. 3B). On the other hand, at the lowest shear stress (0.9 dyn/cm^2^), the maximum distance traveled was only 122.9 µm and the minimum distance traveled remained 10 µm (Fig. 3B). The maximum distance traveled by 3D7-IEs^schi+K^ was longer (430–490 µm) at 4, 3, and 2 dyn/cm^2^ but decreased to 338 and 198 µm at 1.5 and 0.9 dyn/cm^2^, respectively. The minimum distance traveled by 3D7-IEs^schi+K^ remained the same (10 µm) at all shear stresses (Fig. 3C).

**Figure 4:**
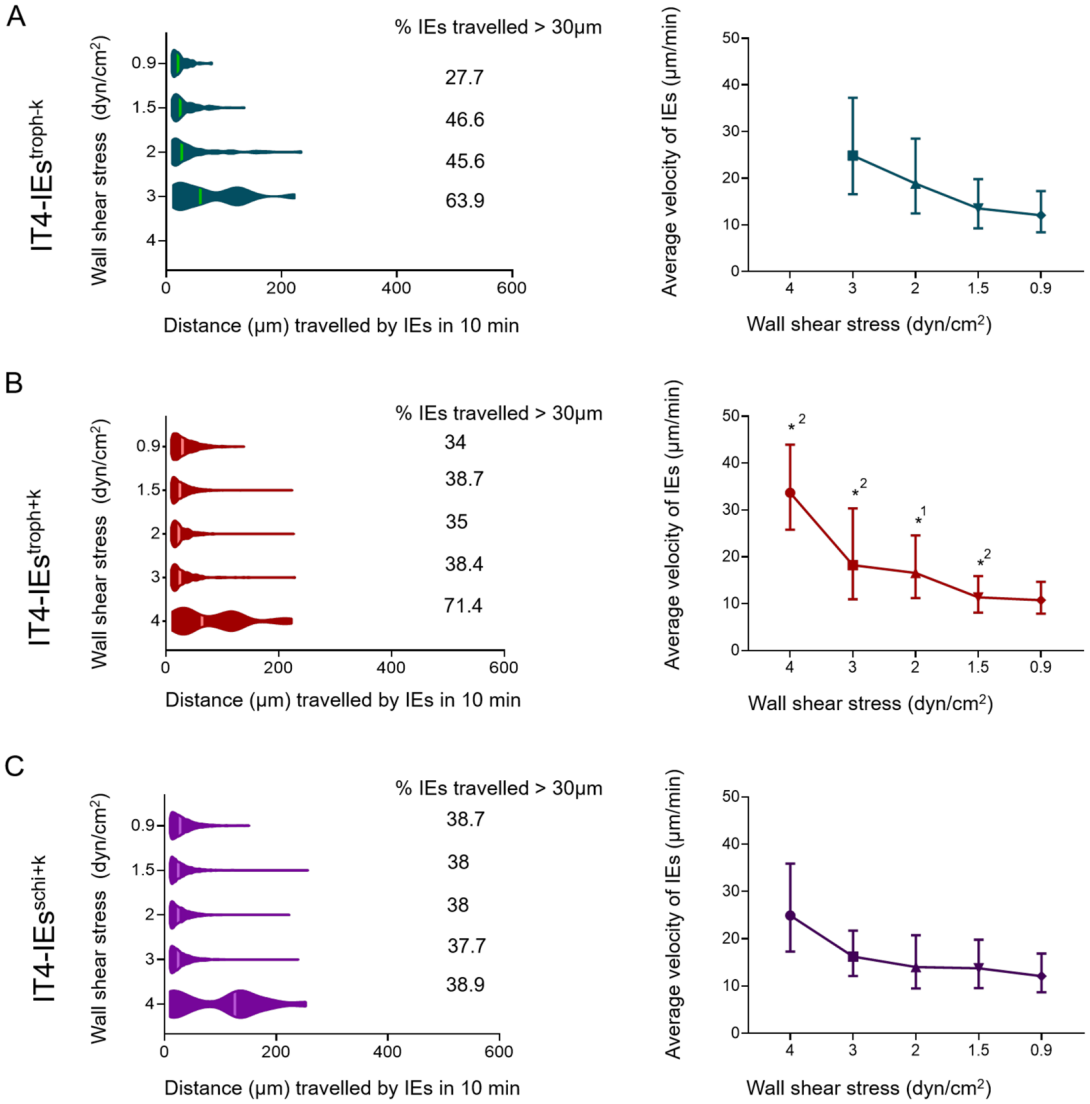
Tracking of IT4-IEs over CHO-745^CD36^ cells. Knobless and knobby trophozoite-stage IT4-IEs (IT4-IEs^troph−K^ (**A**), IT4-IEs^troph+K^ (**B**) and knobby schizont stage IT4-IE^schi+K^ (**C**)) were analyzed. The distance traveled and the average velocity of IEs were calculated using the trackdem R package^38,58^. The distance traveled by each IE was divided by the individual contact time with cells to calculate velocity (n = 20). Significance was assessed using the Kolmogorov-Smirnov test. *P < 0.01 (^1^statistical comparison of IT4-IEs^troph−K^ and IT4-IEs^troph+K^, ^2^statistical comparison of IT4-IEs^troph+K^ and IT4-IEs^schi+K^).

The rolling phenotype was analyzed in detail by calculating the velocity of individual IEs. The average velocity of 3D7-IEs^troph−K^ was 46.41, 30.40, 16.07, and 5.3 µm/min at 3, 2, 1.5, and 0.9 dyn/cm^2^, respectively (Fig. 3A). The average velocity of 3D7-IEs^troph+K^ was 79.2, 26.38, 24.78, 27.30, and 11.94 µm/min at 4, 3, 2, 1.5, and 0.9 dyn/cm^2^, respectively (Fig. 3B). The average velocity of 3D7-IEs^schi+K^ was 19.44, 15.27, 18.66, 17.63, and 13.01 µm/min at 4, 3, 2, 1.5, and 0.9 dyn/cm^2^, respectively (Fig. 4C).

### Effects of the presence of knobs and the parasite stage on the distance traveled by and velocity of IT4-IEs rolling over CHO-745^CD36^ cells

At 3 dyn/cm^2^, IT4-IEs^troph−K^ traveled a minimum distance of 11.9 µm and a maximum distance of 222.7 µm, with a geometric mean of 48.4 µm (Fig. 4A). Although the minimum distance traveled (10 µm) was almost the same at all shear stresses, the maximum distance traveled decreased as shear stress decreased (234, 135, and 79 µm at 2, 1.5, and 0.9 dyn/cm^2^, respectively) (Fig. 4B). IT4-IEs^troph+K^ traveled a maximum distance of 222–228 µm at 1.5–4 dyn/cm^2^ and 138.9 µm at 0.9 dyn/cm^2^ (Fig. 4C). The minimum distance traveled by IT4-IEs^troph+K^ was 10 µm at all shear stresses, except for 4 dyn/cm^2^ where it was 12 µm (Fig. 4B). IT4-IEs^schi+K^ traveled the same minimum distance (10 µm) at all shear stresses (Fig. 4C). The maximum distance traveled by IT4-IEs^schi+K^ was 328.7 µm at 4 dyn/cm^2^, but remained almost constant at 3, 2, and 1.5 dyn/cm^2^ (229, 213, and 247 µm, respectively) (Fig. 4C).

The average velocity of IT4-IEs^troph−K^ was 24.8, 18.82, 13.52, and 12.04 µm/min at 3, 2, 1.5, and 0.9 dyn/cm^2^, respectively (Fig. 4A). The average velocity of IT4-IEs^troph+K^ was 33.66, 18.24, 16.58, 11.38, and 10.76 µm/min at 4, 3, 2, 1.5, and 0.9 dyn/cm^2^, respectively (Fig. 4B). The average velocity of IT4-IEs^schi+K^ was 24.92, 16.24, 14.04, 13.78, and 12.10 µm/min at 4, 3, 2, 1.5, and 0.9 dyn/cm^2^, respectively (Fig. 4C).

### Binding behaviors of receptor-enriched trophozoite-stage IEs to CHO-745^ICAM-1^, CHO-745^P-selectin^, and CHO-745^CD9^ cells

**Figure 5:**
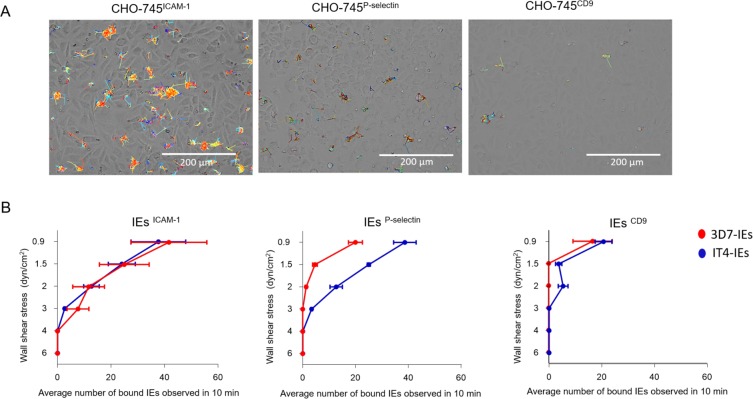
Static adhesion of IEs over ICAM-1, P-selectin, and CD9. The trajectories by IEs over the 3 receptors. (**A**) Average number of bound IEs to the three receptors. (**B**) Cells were cultivated in a µ-slide I 0.8 Luer ibiTreat and the fluidic unit was connected with 1 × 10^7^ IEs. Different shear stresses were applied using the ibidi pump system. Parasites were enriched to bind to the respective receptor via at least seven rounds of selection. The number of adherent IEs was determined after 10 min of flow under various shear stresses (6, 4, 3, 2, 1.5, and 0.9 dyn/cm^2^). Bound IEs were counted in the total image field (0.2 mm^2^) and are represented as mean ± SEM. Examples of selected trajectories are shown S5 Movie.

Analysis of the binding behavior of the IEs to ICAM-1, P-selectin and CD9 revealed very short binding traces inverse to the rolling binding to CD36 (Fig. 5A). IEs tended to cluster in small conglomerates. We concluded that binding to these receptors was stationary (defined as capture of IEs from the flow without rolling or tethering) (S5 Movie). 3D7-IEs^ICAM-1^ showed minimal binding to CHO-745^ICAM-1^ cells at 3 dyn/cm^2^, with 7 ± 7 IEs/0.2 mm^2^. This increased to 11 ± 10, 25 ± 16, and 41 ± 24 IEs/0.2 mm^2^ at 2, 1.5, and 0.9 dyn/cm^2^, respectively (Fig. 5B). 3D7-IEs^P-selectin^ bound to CHO-745^P-selectin^ cells at 2 and 1.5 dyn/cm^2^, with no more than 5 IEs/0.2 mm^2^. This increased to 20 ± 4 IEs/0.2 mm^2^ at 0.9 dyn/cm^2^. Binding of 3D7-IEs^CD9^ to CHO-745^CD9^ cells was less than 5 IEs/0.2 mm^2^ at 2 and 1.5 dyn/cm^2^, but increased to 20 ± 5 IEs/0.2 mm^2^ at 0.9 dyn/cm^2^ (Fig. 5B).

The binding capacity of IT4-IEs to the three ECRs is shown in Fig. 5B. At 6 dyn/cm^2^, IT4-IEs did not bind to CHO-745^ICAM-1^, CHO-745^P-selectin^, or CHO-745^CD9^ cells. IT4-IEs^ICAM-1^ did not adhere until shear stress reached 3 dyn/cm^2^ (2 ± 0.5 IEs/0.2 mm^2^). The number of bound IT4-IEs^ICAM-1^ increased as shear force decreased (12 ± 5, 24 ± 9, and 37 ± 18 IEs/0.2 mm^2^ at 2, 1.5, and 0.9 dyn/cm^2^, respectively) (Fig. 5B). IT4-IEs^P-selectin^ began to bind at 4 dyn/cm^2^ (1 ± 0.5 IEs/0.2 mm^2^). The number of bound IT4-IEs^P-selectin^ was 3 ± 0.5, 12 ± 4, 25 ± 1, and 38 ± 7 IEs/0.2 mm^2^ at 3, 2, 1.5, and 0.9 dyn/cm^2^, respectively (Fig. 5B). IT4-IEs^CD9^ bound to CHO-745^CD9^ cells only at 0.9 dyn/cm^2^ (20 ± 6 IEs/0.2 mm^2^) (Fig. 5B).

As a control, static binding assays were performed for IT4 and 3D7 IEs^troph+K^ over CHO-745^GFP^, where non-specific binding was not observed (Fig. S6B). Additionally, flow experiments were performed with IEs over CHO-745^GFP^ cells. After 30 min at 0.9 dyn/cm^2^, very few bound IEs were detected (3–6 IEs/0.2 mm^2^). Another control was performed by allowing IT4-IEs^ICAM-1^ and 3D7-IEs^ICAM-1^ to bind to CHO-745^CD36^ cells under flow conditions using the same experimental set-up. Interestingly, IT4-IEs^ICAM-1^ showed rolling binding behavior on CD36, as observed with the long-term cultured parasites.

### Binding behaviors to HBEC-5i cells under controlled flow and temperature conditions

**Figure 6:**
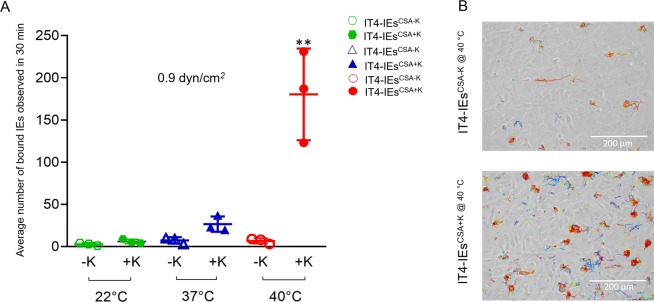
Adhesion of IEs to HBEC-5i cells under flow and controlled temperatures. Average number of bound IEs. (**A**) Trajectories generated by bound IT4-IEs^CSA+K@40 °C^ and IT4-IEs^CSA+K@37 °C^ at 0.9 dyn/cm^2^. (**B**) Experiments were performed using IT4-IEs enriched over HBEC-5i cells for at least seven rounds of enrichment. HBEC-5i cells were cultivated in a µ-slide I 0.8 Luer ibiTreat and the fluidic unit was connected with 1 × 10^7^ IEs. Different shear stresses were applied using the ibidi pump system. Temperature was controlled using a hood over the microscope. An example is shown in S7 and S8 Movies. The number of bound IEs was counted using the trackdem R package^38,58^ in the total image field (0.2 mm^2^) and is represented as the mean ± SEM (**p < 0.001).

To analyze the binding behavior of IT4-IEs to human ECs, we used the HBEC-5i cell line. These cells express CSA on their surface due to immortalization processes, which lead to excessive surface production of CSA^39^. IT4-IEs were enriched for binding to HBEC-5i cells via at least seven rounds of selection to generate a CSA-binding parasite population (IT4-IEs^CSA^). Exclusive expression of *var2csa* was confirmed by qPCR and transcriptome analysis (Fig. S6A)^36^. To select IT4-IEs^CSA^ with knobs (IT4-IEs^CSA+K^), seven rounds of enrichment were performed at 40 °C (Fig. 6A,B), which exclusively selects IT4-IEs^CSA+K ^^36^. Flow experiments with IT4-IEs^CSA−K^ and IT4-IEs^CSA+K^ were performed at room temperature (22 °C), body temperature (37 °C), and febrile temperature (40 °C). Binding was only observed at 0.9 dyn/cm^2^; therefore, we tracked IEs under this shear stress for 30 min. IT4-IEs^CSA−K^ and IT4-IEs^CSA+K^ only statically adhered to CSA on HBEC-5i cells (Fig. 6A,B; S7 and S8 Movies). Figure 6A shows the average numbers of bound IT4-IEs^CSA−K^ and IT4-IEs^CSA+K^ at the three temperatures. At 22 °C, IT4-IEs^CSA−K^ had a very low binding capacity, with an average of 3 ± 1 IEs/0.2 mm^2^, while the average number of bound IT4-IEs^CSA+K^ was 6 ± 1 IEs/0.2 mm^2^. Increasing the temperature to 37 °C only increased the binding of IT4-IEs^CSA+K^, with an average of 26 ± 5 IEs/0.2 mm^2^. At febrile temperature (40 °C), binding of IT4-IEs^CSA+K^ significantly increased to an average of 180 ± 30 IEs/0.2 mm^2^. Statistical significance was done using ANOVA between the binding capacities at the three different temperature values (*p* = 0.0036).

### Rolling adhesion over CHO-745^CD36^ cells under controlled flow and temperature conditions

**Figure 7:**
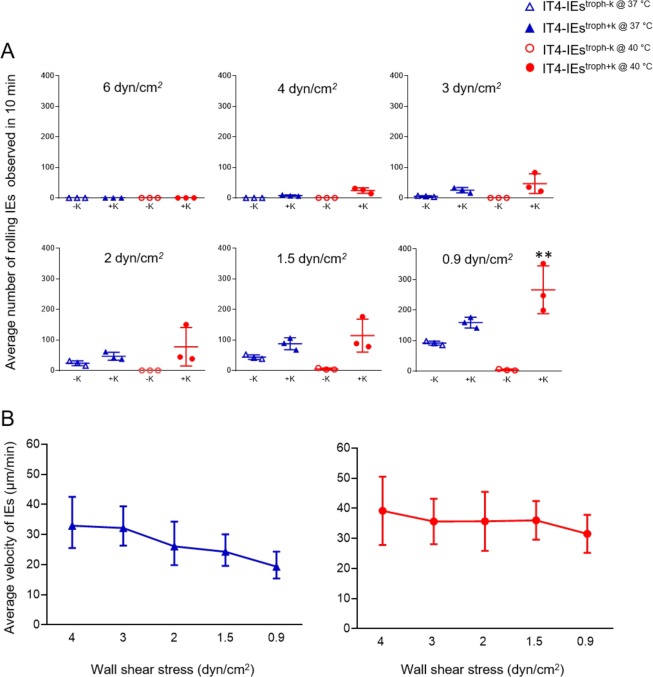
Rolling adhesion behavior of knobless and knobby trophozoite-stage IEs over CHO-745^CD36^ under controlled temperatures. Average number of IEs adhering to CHO-745^CD36^ cells at 37 °C (blue) and 40 °C (red). (**A**) Cells were cultivated in a µ-slide I 0.8 Luer ibiTreat and the fluidic unit was connected with 1 × 10^7^ IEs. Different shear stresses were applied using the ibidi pump system. Binding to cells was analyzed with long-term cultures. The number of adherent IEs was determined after flow for 30 min at 0.9 dyn/cm^2^. The number of rolling IEs was counted using the trackdem R package^38,58^ in the total image field (0.2 mm^2^) and is represented as mean ± SEM (**p < 0.001). (**B**) Average velocity of IT4^troph+K@37 °C^ and IT4^troph+K@40 °C^, respectively. The distance travelled by each IE was divided by the individual contact time with cells to calculate velocity (n = 20).

Next, we analyzed the rolling behavior of IT4-IEs^troph−K^ and IT4-IEs^troph+K^ at different temperatures (37 °C and 40 °C). Figure 7A shows the average number of rolling IEs at 4 dyn/cm^2^. Rolling of IT4-IEs^troph−K@37°^ and IT4-IEs^troph−K@40 °C^ was not observed. On the other hand, IT4-IEs^troph+K@37 °C^ and IT4-IEs^troph+K@40 °C^ rolled, with an average of 7 ± 1 and 34 ± 11 IEs/0.2 mm^2^, respectively. Decreasing shear stress to 3 dyn/cm^2^ increased the average number of rolling IT4-IEs^troph+K@37 °C^ and IT4-IEs^troph+K@40 °C^ to 25 ± 4 and 46 ± 18 IEs/0.2 mm^2^, respectively (Fig. 7A). The average number of rolling IEs was further increased at 2 dyn/cm^2^, with an average of 23 ± 4 IEs/0.2 mm^2^ for IT4-IEs^troph−K@37°^ and 46 ± 7 IEs/0.2 mm^2^ for IT4-IEs^troph+K@37 °C^. Rolling of IT4-IEs^troph−K@40 °C^ was not observed. On the other hand, IT4-IEs^troph+K@40 °C^ rolled with an average of 77 ± 36 IEs/0.2 mm^2^. At 1.5 dyn/cm^2^, the average number of rolling IT4-IEs^troph−K@37 °C^, IT4-IEs^troph+K@37 °C^, IT4-IEs^troph−K@40 °C^, and IT4-IEs^troph+K@40 °C^ was 43 ± 4, 87 ± 11, 4 ± 1, and 114 ± 31 IEs/0.2 mm^2^, respectively (Fig. 7D). Finally, at 0.9 dyn/cm^2^, the number of rolling IT4-IEs^troph−K@37 °C^, IT4-IEs^troph+K@37 °C^, IT4-IEs^troph−K@40 °C^, and IT4-IEs^troph+K@40 °C^ was 91 ± 3, 158 ± 10, 4 ± 1, and 266 ± 45 IEs/0.2 mm^2^, respectively (Fig. 7A).

Tracking of rolling IEs showed that the average velocity of IT4-IEs^troph+K@37 °C^ was 33.98, 32.8, 26.94, 24.83, and 19.82 µm/min at 4, 3, 2, 1.5, and 0.9 dyn/cm^2^, respectively (Fig. 7B). On the other hand, the average velocity of IT4-IEs^troph+K@40 °C^ was 39.19, 35, 35, 36.02, and 31.53 µm/min at 4, 3, 2, 1.5, and 0.9 dyn/cm^2^, respectively (Fig. 7B).

We also observed that, on performing the flow assays at 22 and 37 °C at lower shear stresses (1.5–0.9 dyn/cm^2^) some of the rolling IEs stopped rolling and adhere to CD36 (about 60%). Whereas, this was not observed as we performed the assays at 40 °C, where a lower number of IT4-IEs (3–9 IEs/0.2 mm^2^ < 1%) adhered at all shear stresses.

## Discussion

We developed a new approach to study the binding behavior of IEs. This method integrates a flow system, which mimics vascular mechanobiology under physiological and febrile temperatures, and bioinformatics image tracking procedures to accurately characterize the cytoadhesive behavior of IEs. Wall shear stress *in vivo* varies along the venular tree as vessel diameter and flow rate change. Therefore, we performed experiments under six magnitudes of shear stress. We demonstrated that IEs interacting with CD36 performed rolling adhesion under different shear stresses and at different temperatures. Additionally, the rolling behavior of disc-shaped trophozoite-stage IEs (flipping) differed from that of round-shaped schizont-stage IEs (smooth continuous rolling). Studies by Fedosov and colleagues using multiscale IE modeling are consistent with this finding^20,23^. However, binding behavior to CD36 is controversial. In agreement with the current study, Herricks and colleagues observed rolling adhesion of isogeneic IEs over a microfluidic slide coated with CD36^40^. Rolling adhesion was also observed over human dermal microvascular ECs, which express numerous CD36 molecules on their surface (86 ± 14 × 10^6^ molecules/mm^2^)^41^. Lansche and colleagues reported that even deformed trophozoite-stage IEs with sickle cell traits perform rolling adhesion over human dermal microvascular ECs^42^. A chimeric SCID mouse model was used to mimic the human microvasculature *in vivo*. The authors also observed rolling adhesion performed of patients’ IEs on human skin grafts with CD36 on their surface^19^. On the other hand, other studies suggested that IEs only bind statically to CD36^16,18^. CD36 is the main interacting partner of IEs because more than 40 of the 60 *Pf*EMP1 family members contain a CD36-binding domain (CIDRα2–6)^35,43,44^. This supports the theory that *Pf*EMP1-CD36 interactions are essential for survival of the parasite in its host.

CD36 is a class B scavenger transmembrane glycoprotein found on the microvascular endothelium, macrophages, microglia, adipocytes, and platelets^45^. This receptor binds to a diverse range of ligands including pathogen-associated molecular patterns and modified self-molecules^46^. CD36 forms a ‘hairpin-like’ structure with a large ectodomain and two transmembrane domains^45^. The ectodomain of CD36 is capped by a three-α-helix bundle and an apex region that contains an accumulation of cationic residues, which are proposed to form a binding site for polyanionic ligands. Co-crystallization studies of CIDRα2-6 domains and the CD36 ectodomain identified a small conserved hydrophobic pocket in CIDRα peptides that forms a non-covalent hydrogen bond with a complementary hydrophilic phenylalanine residue (F153) in CD36. This combination generates a high affinity and stable binding site that allows the domains to interact with their ligands with a slow off rate, which stabilizes cytoadhesion of IEs against the strong forces generated by blood flow^47^. In the current study, cytoadhesive dynamics to CD36 differed according to the conditions. The absence of knobs on the surface of IEs shortened the rolling distance, whereas the presence of knobs led to maintenance of the rolling pattern for longer distances in the case of both trophozoite- and schizont-stage IEs. We also observed that the distance travelled by the IEs at higher shear stresses in both isolates have two peaks. This might be due to the control of the shear stresses to the rolling movement of the IEs. Some *Pf*EMP1 molecules on the surface of IEs might perform a stronger bond with CD36, so that the IEs resist flow while others with weaker bond might not be able to roll longer distance and will be washed with the flow.

The mean rolling velocity of IEs over CD36 was different between different stages. We observed that schizonts roll more stable at different shear stresses than trophozoites. The flipping of trophozoites was quicker at higher shear stresses than the lower ones. In this study, all the recorded velocities at different stages and conditions were less than 1 µm/sec which was not recorded by Antia and colleagues. In their study, they observed higher velocities few to tens µm/sec. This might be due to the fact that they performed the assays over recombinant proteins (CD36 and ICAM-1) which might change the binding behavior^16^.

Comparing the mean rolling velocities of the knobby IT4-IEs at 37 °C versus 40 °C showed no statistical significance, in both cases they roll with a mean velocity of 30–50 µm/min which is higher than the mean velocity at room temperature (30–20 µm/min). This difference highlights the effect of temperature on binding dynamics between CD36 and *Pf*EMP-1.

Shear stress significantly affected the velocity of IEs without knobs on their surface. It seems that flow influenced IEs lacking knobs. This might be because the density of *Pf*EMP1 is supposed to be lower on the surface of IEs without knobs than on the surface of IEs with knobs^48^. On the other hand, tethering and receptor attachment of IEs with knobs seems to be controlled independently of shear stress. This was observed from the stable mean of velocity observed during the experiments performed with IEs^+k^ (Figs. 3 and 4). Thus, the presence of knobs benefits parasites by stabilizing the ligand-receptor interaction due to the concentrated amount of *Pf*EMP1 on the knob’s surface. Slip binding occurs between CD36 and *Pf*EMP1, and its life time is decreased by external forces (e.g., shear stress)^34^, which might explains the rolling behavior on higher shear stresses and static binding on the lower ones. Interestingly, rolling of schizont-stage IEs was more stable and continued for longer distances than that of trophozoite-stage IEs. The surface of schizont-stage IEs is homogenously covered by knobs, which is assumed to slow down rolling movement^41^. In addition, the adhesion force is smaller at the schizont stage than at the trophozoite stage, despite the high concentration of adhesion proteins in the former stage^49^.

We made the following findings regarding binding of IEs to ICAM-1, P-selectin, and CD9: (i) binding occurs mostly at lower shear stresses (starting from 2 dyn/cm^2^), (ii) IEs mostly bind in a conglomerate pattern and resist flow, and (iii) no rolling pattern is observed. This suggests that catch bonds mediated binding. These are defined as non-covalent bonds that have increased lifetimes only when external force is applied^27,28^. On the other hand, IT4-IEs^ICAM-1^ showed rolling binding behavior on CD36, this IEs population is known to express mainly IT4-*var*01, this encodes for a protein which have both head structure for CD36 binding and also an ICAM-1 binding domain^33^. In accordance with the current study, such static binding to ICAM-1 was previously reported^19^. The low binding capacity at higher shear stress might be due to the lack of CD36 on the surface of CHO-745 cells. We believe that CD36 is the main receptor responsible for tethering of IEs and allows them to roll slowly before being immobilized by other ECRs. In addition, the presence of knobs did not affect the binding capacity or pattern to ICAM-1, P-selectin, and CD9, suggesting that these receptors play a secondary role in sequestration of IEs and only when ECs are stimulated with inflammatory cytokines. Previous studies provided more details about the binding dynamics of ECs to different receptors (including selectins and ICAM-1). When an activation signal is detected, ECRs switch from a low to a high affinity state due to changes in bond formation rates and bond dissociation properties^50^.

We investigated the thermodynamics of IE-ECR interactions. Most of these interactions occur at body temperature (37 °C) and during fever (up to 40 °C). We used HBEC-5i cells, which express CSA on their surface. Our flow experiments demonstrated the importance of knobs for parasite survival. Only IEs with knobs could withstand the high temperature and bind under shear stress of 0.9 dyn/cm^2^. In addition, rolling adhesion to CD36 was preserved and the number of rolling IEs was increased at 40 °C and under higher shear stress (starting from 4 dyn/cm^2^). Normally, fever is a reaction to a pathogen in order to protect the host^27,50^. Fever induces expression of adhesive receptors (i.e., ICAM-1) on the surface of ECs and leukocyte trafficking^27^, but parasites are well prepared for these changes. Carvalho and colleagues measured the cytoadhesive force of IEs to CSA via force spectroscopy. They found that the binding force is notably decreased at febrile temperature, but the number of bound IEs increases^51^. They speculated that this increase in binding despite the decrease in force is due to non-specific binding. Another study demonstrated that, the binding affinities to both CD36 and ICAM-1 were decreased at febrile temperature (41 C°)^34^. The difference between our experiments and those performed by Lim and colleagues that^34^, they used recombinant proteins (ICAM-1 and CD36) in their binding studies and we used transfected CHO-cells that express the receptor of interest. We do believe that these two methods lead to different binding phenotypes between the receptor and ligand. On the other hand, it might be also that other proteins on the surface of the cells might help in increasing the binding capacity (for example heat shock proteins), but it will be difficult in the current study to confirm this explanation.

In conclusion, our data completely describe the cytoadhesive dynamics between IEs and ECRs in the presence and absence of knobs. IEs are first captured from the circulation via CD36, and this is followed by tethering and slow rolling. Other ECRs are expressed as the parasite load increases, which enhances immobilization of IEs to the surface of ECs. We also highlighted the importance of knobs for the thermodynamics of IE-ECR interactions. Higher temperatures also affect the binding dynamics between IEs and ECRs: (i) increased binding of knobby IEs to CSA, (ii) increased number of knobby IEs rolling over CD36, (iii) decreased number of immobilized knobby IEs over CD36.

## Methods

### Parasite culture

IT4 (FCR3S1.2) and 3D7 *P. falciparum* isolates were cultivated with human O^+^ erythrocytes (5% hematocrit; UKE, Hamburg, Germany) in the presence of 10% human serum A^+^ (Interstate Blood Bank, Inc. Memphis, TN, USA) according to standard procedures^52^. Parasite cultures were synchronized once per week using 5% sorbitol^53^.

### Transfection and culture of CHO-745 cells

CHO-745 cells defective in glycosaminoglycan biosynthesis (American Type Culture Collection (ATCC); no. CRL-2242) were transfected and cultured as previously described^8,31,33^. CHO-745 cells transfected with GFP, CD36, ICAM-1, P-selectin, and CD9 were used. These five cell lines were routinely sorted for surface expression of the receptors via fluorescence-activated cell sorting.

### Selection and enrichment of IEs that bind to ECRs of interest

As previously described^33^, IEs (10% parasitaemia) were pre-absorbed over CHO-745^GFP^ cells for 60 min at 37 °C in binding medium (RPMI containing 2% glucose, pH 7.2). Thereafter, non-binding IEs were incubated with transfected CHO-745 cells for 60 min at 37 °C. Washing was then performed using binding medium for 7 times and then controlled under inverted microscope for any unbound IEs. Bound IEs were cultivated up to a parasitaemia of 10%. The entire enrichment procedure was repeated weekly for 7 weeks.

### Selection and enrichment of IEs that bind to HBEC-5i cells

For enrichment of IEs for binding to HBEC-5i, the same procedure as described above was performed. At least seven rounds of selection were performed at 37 °C and 40 °C^36^. The HBEC-5i cell line was cultivated according to ATCC guidelines.

### Enrichment of knob-containing IEs using gelatin flotation

IEs were resuspended in two volumes of 1% pre-warmed gelatin (175 g Bloom, Sigma-Aldrich, Germany) and incubated for 45 min at 37 °C^54^. The supernatant, which harbored knob-containing IEs, was washed with RPMI and cultivated as usual. This procedure was repeated each week. The presence of knobs was confirmed by electron microscopy.

### qPCR

As a control, expression levels of *var* genes and *kahrp* in each parasite population were determined by qPCR. qPCR was performed using sense and antisense primers designed to amplify 100–120 bp fragments of the respective genes (S8 Text). After RNA isolation, cDNA was synthesized using SuperScript II Reverse Transcriptase (Invitrogen, Germany) and random hexamers (Invitrogen) at 50 °C for 1 h. The cDNA template was mixed with SYBR Green PCR Master Mix (QuantiTect SYBR Green PCR Systems, Qiagen, Germany) and 0.5 µM forward and reverse primers in a final volume of 10 µl. Samples were incubated at 95 °C for 15 min, and then subjected to 40 cycles of 95 °C for 15 sec and 60 °C for 1 min, followed by a melting step (60–95 °C). The specificity of each primer pair was confirmed after each qPCR run by performing dissociation curve analysis. *var* gene expression was normalized against expression of the conserved ring-stage gene *Skeleton-binding protein 1* (*sbp1)* and the housekeeping genes *Fructose-bisphosphate aldolase* and *arginyl-tRNA*. Expression of each *var* gene was calculated relative to the geometric mean expression of these three normalizers^55,56^.

### Electron microscopy

Trophozoite-stage *P. falciparum* cultures were separated by Percoll density gradient centrifugation and fixed with 2.5% glutaraldehyde and 2.5% paraformaldehyde (Electron Microscopy Science, Hatfield, PA, USA). Samples were washed with 50 mM sodium cacodylate trihydrate buffer, pH 7.4 (Electron Microscopy Science), and post-fixed with 2% OsO_4_ prepared in H_2_O for 45–60 min on ice in the dark. After heavy metal staining using 0.5–1% uranyl acetate (Electron Microscopy Science) for 45–60 min at room temperature, samples were dehydrated using an increasing ethanol series prepared in H_2_O. Embedding and polymerization were performed using increasing concentrations of epoxy resin (EPoN 812; Carl Roth, Karlsruhe, Germany) at 60 °C. Polymerized samples were cut into 55–60 nm sections (Ultracut UC7, Leica, Germany), placed on 300 mesh copper grids, and analyzed with a transmission electron microscope (Tecnai Sprit TEM, FEI, Netherland) at an acceleration voltage of 80 kV. By counting 150 IEs from each sample, the percentage of knobby to knobless IEs was determined.

### Binding experiments under flow conditions

1. Seeding of CHO cells. A total of 1.5 × 10^5^ CHO cells were seeded into a µ-slide I 0.8 Luer ibiTreat in 200 µl Ham’s F12 medium containing G418 as a selection marker 2 days before the assay. Cells were 90–100% confluent on the day of the assay.
2. Enrichment of IEs containing parasites at the trophozoite or schizont stage by magnetic-activated cell sorting. On the day of the assay, IEs containing trophozoites (28–32 hpi) or schizonts (36–44 hpi) were enriched using various MACS magnetic separation columns as described previously^57^. Briefly, cultures were centrifuged, resuspended in binding medium such that hematocrit was 10%, and loaded onto the top of the column. The outflow, which contained non-IEs and ring-stage and young trophozoites, was discarded. Finally, trophozoite- or schizont-stage IEs were eluted in binding medium and incubated over CHO-745^GFP^ cells for 60 min at 37 °C^33^. Finally, unbound cells were washed with binding medium and the cell count was adjusted to 1 × 10^7^ using a Neubauer chamber.
3. Flow assay set-up: a unidirectional laminar flow system (ibidi pump system) was used. According to the manufacturer’s instructions, IEs obtained in step 2 were resuspended in binding medium and added to the fluidic unit of the flow system. The fluidic unit was then connected to the CHO-745 cell slide and pump system. A range of wall shear stresses similar to those detected in venular microcapillaries^26^ were applied using software to control the pump. Specifically, 6, 4, 3, 2, and 1.5 dyn/cm^2^ was applied for 10 min, and 0.9 dyn/cm² was applied for 30 min. Imaging was performed using an EVOS FL Auto inverted microscope (Thermo Fisher Scientific, Waltham, USA). An image was acquired every 5 sec using a control program. The experiment was started by simultaneously switching on the pump system control program and the microscope control program.

### Bioinformatics to track IEs and analyze trajectories

The trackdem (0.4.2) R package was used to characterize the behavior of IEs^38^. In each experiment, the image sequence recorded under each shear stress was loaded in the R‐environment (3.5.1)^58^. A background image containing all motionless objects was created. Moving particles were detected by subtracting this background from all images. Identification was optimized using machine learning. The Shiny R package was used with thresholding of 0.1. In the *PixelRange* argument, a range of 30–500 was used to filter any unwanted noise. Particle tracking was then performed to generate tracked segments connecting the moving IEs in each frame. Individual trajectories were subsequently reconstructed based on a minimum present *(incThres*) equal to six frames (i.e., 30 sec). The reconstructed trajectories were plotted as images and presented in AVI animations. The accumulated distance covered by each IE was calculated by multiplying the total displacement in pixels by 0.4572. The detailed R-script used is provided in S10 Text. To calculate the average velocity of each individual IE, the accumulated distance was divided by the calculated contact time with cells. IEs that traveled less than 50 µm were excluded from the analysis.

### Robustness checks

Manual tracking was performed and compared with automated tracking. ImageJ (Version 1.48 V, National Institutes of Health, Maryland, USA) was used to evaluate each experiment. The accumulated distance traveled by individual IEs was tracked using the Manual Tracking and Chemotaxis plugins.

### Statistical analysis

Geometric mean was calculated using GraphPad Prism 8. Statistical significance was assessed using the Kolmogorov-Smirnov test in flow experiments and the Kruskal Wallis test in qPCR experiments (GraphPad Prism 8).

## Supplementary information

Supplemental Information 1.

Supplemental Information 2.

Supplemental Information 3.

Supplemental Information 4.

Supplemental Information 5.

Supplemental Information 6.

Supplemental Information 7.

Supplemental Information 8.
