# Supplementary figures and images for: Adhesion between P. falciparum infected erythrocytes and human endothelial receptors follows alternative binding dynamics under flow and febrile conditions
Source: Sci Rep. 2020 Mar 11;10:4548. doi: 10.1038/s41598-020-61388-2 (PMC7066226; doi:10.1038/s41598-020-61388-2)

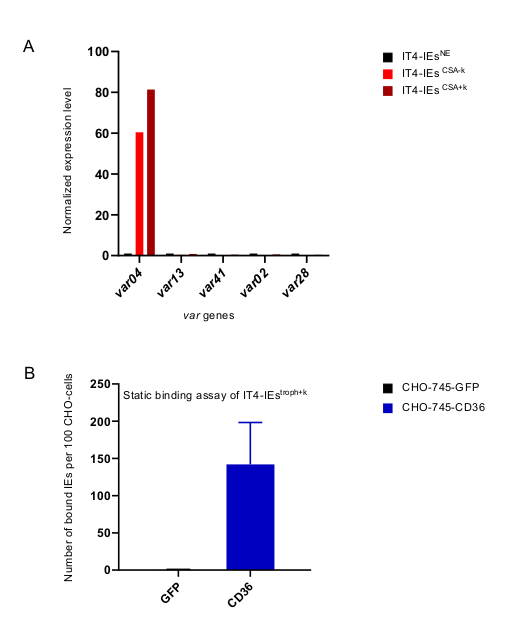

Supplement: Supplementary file 5 — Supplemental Information 5. [file 41598_2020_61388_MOESM5_ESM.tif]
